# *Ampelocissus asekii* J. Wen, R. Kiapranis & M. Lovave, a new species of Vitaceae from Papua New Guinea

**DOI:** 10.3897/phytokeys.21.4512

**Published:** 2013-03-15

**Authors:** Jun Wen, Robert Kiapranis, Michael Lovave

**Affiliations:** 1 Department of Botany, National Museum of Natural History, MRC 166, Smithsonian Institution, Washington, D.C. 20013-7012, USA; 2 Forest Biology Program, PNG Forest Research Institute, P.O Box 314, Lae 411, Morobe Province, Papua New Guinea

**Keywords:** *Ampelocissus*, *Ampelocissus asekii*, Morobe Province, Papua New Guinea, Vitaceae

## Abstract

A new species *Ampelocissus asekii* J.Wen, R.Kiapranis & M.Lovave of Vitaceae is described from Morobe Province of Papua New Guinea. It is a close relative of *Ampelocissus muelleriana* Planch., another endemic of New Guinea and differs from the latter by its densely woolly tomentose lower leaflet surface and much thicker leaflets. The new species is from the mid montane forests, whereas *Ampelocissus muelleriana* occurs in the lowland rain forests.

## Introduction

*Ampelocissus* Planch. (Vitaceae) consists of ca. 95 species from tropical Asia, Africa, Australia and Central America (Wen 2007). The genus was established by Planchon in 1884 and is characterized by the inflorescences subtended by a tendril, bisexual flowers, and a floral disc usually with ten linear marks on its side (Planchon 1887). Lauterbach (1925) recognized two species of the genus in New Guinea, including the simple-leaved *Ampelocissus arachnoidea* Planch. and the trifoliate *Ampelocissus muelleriana* Planch. A third species *Ampelocissus acetosa* (F. Muell.) Planch. with pedate leaves has been recorded from Papua New Guinea (Jackes 1984). Nevertheless no specimens of *Ampelocissus arachnoidea* have been examined by the authors. Lauterbach's locality of the species “Key-Inseln: Doela” is probably either Kai (Kei ) Dulah or Dulah Laut in the Kai (Kei) Islands of Indonesia, off the southwest coast of New Guinea. We herein report a new species from Morobe Province based on our herbarium studies.

## Methodology

Herbarium work was conducted in the Papua New Guinea National Herbarium inLae (LAE). Micromorphological characters were documented with scanning electron microscopy (SEM). The SEM samples were coated with gold and palladium, and observed under a Zeiss EVO M15 electron microscope with LaB6 electron source at the SEM Lab of the National Museum of Natural History, the Smithsonian Institution.

## Results and discussion

### Ampelocissus asekii

J. Wen, R. Kiapranis & M. Lovave sp. nov.

urn:lsid:ipni.org:names:77125856-1

http://species-id.net/wiki/Ampelocissus_asekii

Figs 1 [Fig F2] –3

#### Type.

Papua New Guinea. Morobe Province: near Haumga, Aseki Patrol area, steep gully in secondary lower montane forest, alt. 4600 ft, liana, leaves glossy mid green above, brownish below, buds dark brown, 6 Apr 1966, L. A. Craven & R. Schodde 1219 (holotype: LAE!, accession number 143956; isotypes: A, CANB, K, L).

#### Description.

Liana. Young stems with dense light-brown woolly hairs. Leaves trifoliate; stipules narrowly triangular, 3–5 mm long, 2–3.5 mm wide; leaflets chartaceous, woolly pubescent with white hairs above, densely rusty woolly pubescent below, base of lateral leaflet strongly oblique, that of terminal leaflet acute, but slightly oblique, apex acute, margin sinuate-spinulose with 3–10 teeth on each side; petiole 9.5–10.5 cm long, densely woolly pubescent; blade of terminal leaflets obovate, blade of lateral leaflets unequally ovate, 13–15 cm long, 6–8 cm wide; lateral veins 5–7 on each side; petiolule 2–3 cm long, densely woolly pubescent. Tendril simple. Inflorescence ca. 35 cm long, a narrow panicle of spikes with all but one branch aggregated on the top 1/3 of the inflorescence primary axis, primary branches ca. 35, each 1–2.8 cm long, light rusty woolly pubescent, each with 6–25 flowers; peduncle ca. 10 cm long. Flowers sessile, 4 to rarely 5-merous; calyx saucer-shaped, glabrous, 0.2–0.3 mm long, 0.5–0.6 mm wide; petals oblong to ovate-oblong, glabrous, 1.0–1.3 mm long, 0.5–0.7 mm wide; floral disc adnate to ovary, grooved; stamens with filaments 0.5–0.6 mm long and ovate-oblong anthers; ovary 2-locular, styles short, 0.2–0.3 mm long. Fruits not seen.

**Figure 1.**
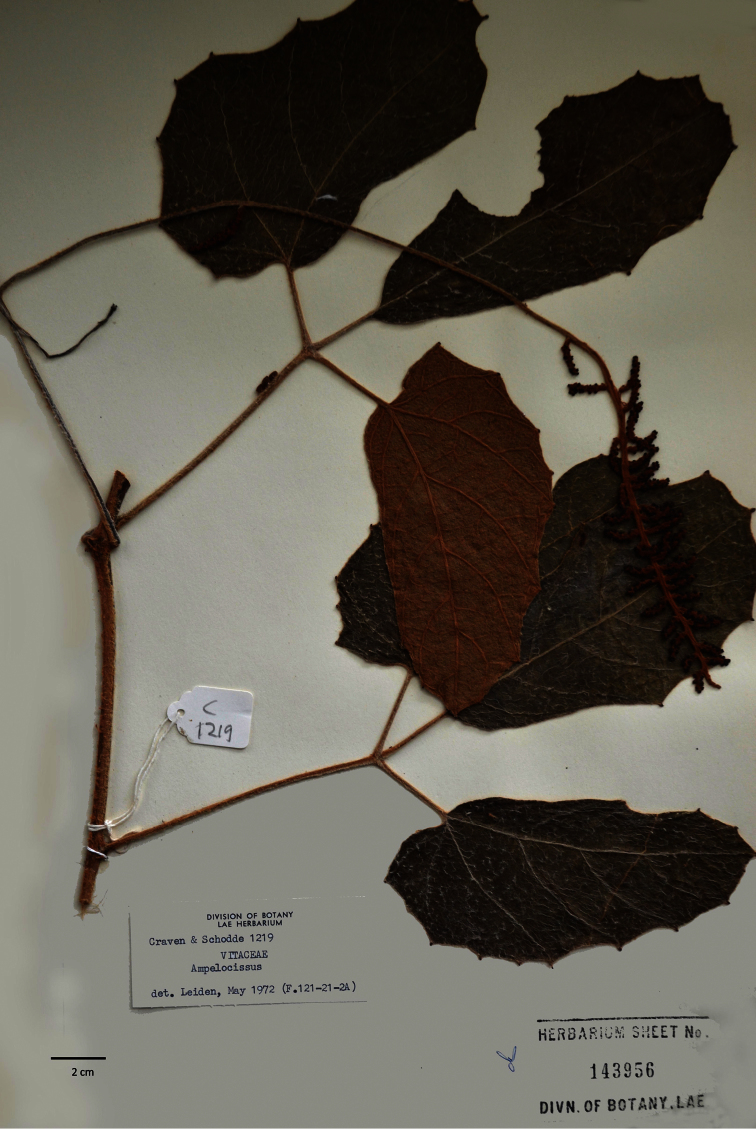
Image of the holotype of *Ampelocissus asekii* J.Wen, R.Kiapranis & M.Lovave.

**Figure 2. F2:**
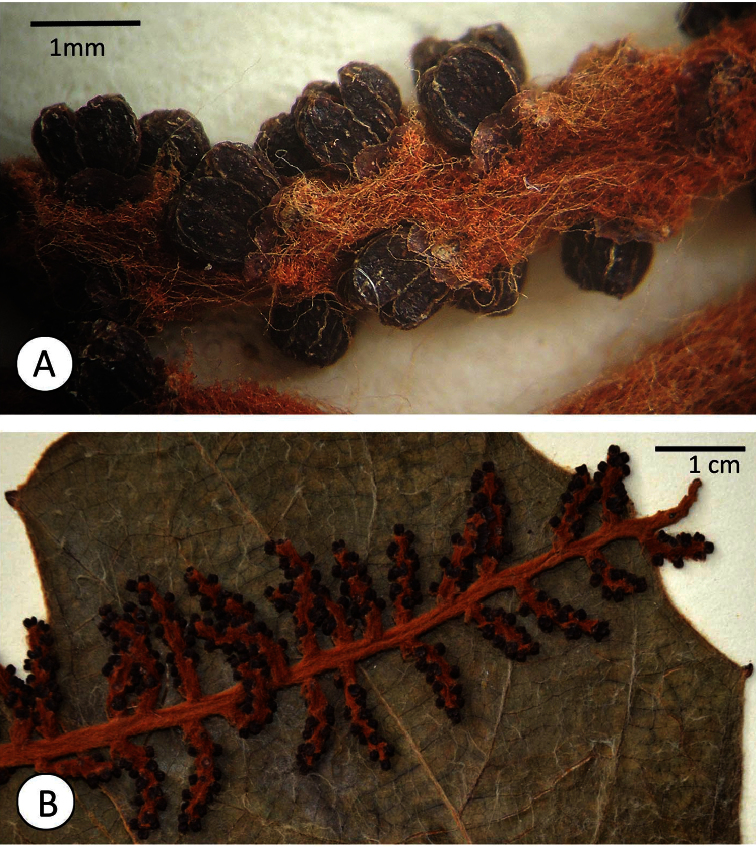
*Ampelocissus asekii* J.Wen, R.Kiapranis & M.Lovave. **A** Close-up of an inflorescence branch showing trichomes and floral morphology **B** A portion of inflorescence laying on the adaxial leaflet surface.

**Figure 3.**
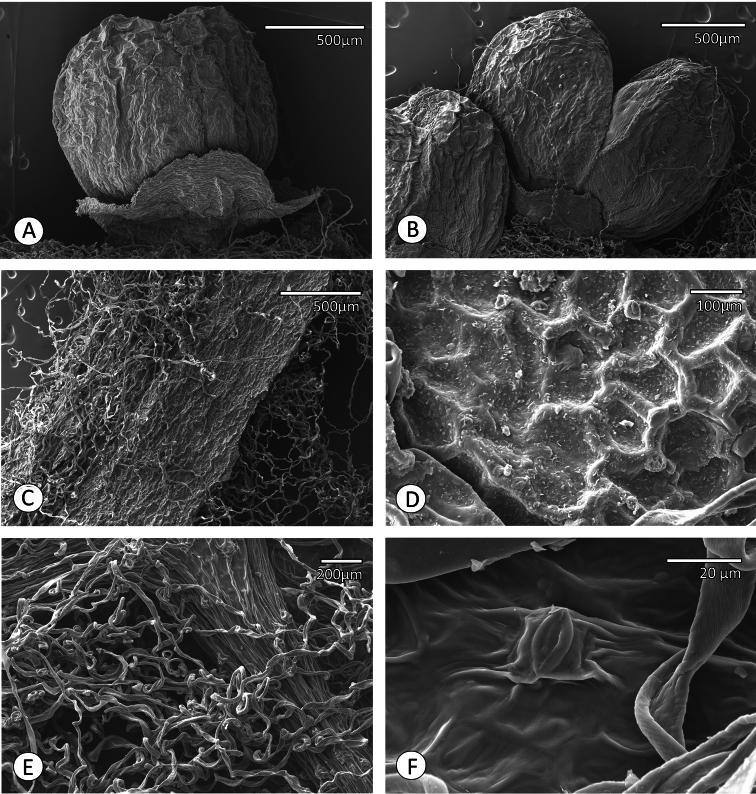
Micromorphology of *Ampelocissus asekii* J.Wen, R.Kiapranis & M.Lovave. **A** Floral bud showing sepals and petals **B** An opening floral bud **C** Trichomes on adaxial leaflet surface near a veinlet **D** Cuticle on adaxial leaflet surface **E** Trichomes on abaxial leaflet surface **F** Cuticle and a stoma on abaxial leaflet surface.

#### Distribution.

The new species is only known from its type locality in Morobe Province, Papua New Guinea.

#### Ecology.

Mid montane forests. Fl. Apr; elevation at ca. 1500 m.

#### Etymology.

This species is named after the type locality, the Aseki Patrol area in Morobe Province.

The new species is closely related to *Ampelocissus muelleriana*, another endemic from New Guinea. They differ in the highly distinctive brown woolly hairs on the lower leaflet surface, the acute leaflet apex and the chartaceous leaflet texture in the new species (vs. with tufts of rusty hairs in the axils of veins on the lower surface, acuminate leaflet apex, and thin chartaceous to membranaceous leaflet texture in *Ampelocissus muelleriana*). The new species occurs in the mid montane zone, whereas *Ampelocissus muelleriana* grows in the lowland rain forests in East Sepik, Madang and Morobe of Papua New Guinea as well as in West Papua of Indonesia.

##### Below we provide a key to all four *Ampelocissus* species in New Guinea

**Table:** 

1a	Leaves simple or pedately compound, inflorescence a thyrse with pedicellate flowers	2
2a	Leaves simple	*Ampelocissus arachnoidea*
2b	Leaves pedate with (5-) 7-9 (-11) leaflets	*Ampelocissus acetosa*
1b	Leaves trifoliate, inflorescence a panicle with spicate inflorescence units (sessile or nearly sessile flowers)	3
3a	Lower leaflet surface densely rusty woolly pubescent, leaflet chartaceous in texture, in mid montane habitat	*Ampelocissus asekii*
3b	Lower leaflet surface with rusty hairs at the leaflet vein axils, leaflet thin chartaceous or membranaceous; in lowland rain forest habitat	*Ampelocissus muelleriana*
*Ampelocissus arachnoidea* is tentatively included in the key.		

## Supplementary Material

XML Treatment for Ampelocissus asekii
